# Intermittent fasting for rheumatic diseases: a systematic review and meta-analysis of conflicting evidence from observational studies and randomized controlled trials

**DOI:** 10.7717/peerj.21185

**Published:** 2026-04-27

**Authors:** Ruofan Liu, Chuanwei Zhang, Yunfei Li, Chuanbing Huang

**Affiliations:** The First Affiliated Hospital of Anhui University of Chinese Medicine, Hefei, Anhui, China

**Keywords:** Intermittent fasting, Rheumatic diseases, Rheumatoid arthritis, Meta-analysis, Disease activity

## Abstract

**Background and Objectives:**

Intermittent fasting (IF) has emerged as a dietary approach with potential therapeutic effects that is gaining recognition. Although several studies have explored the impact of IF on clinical symptoms in patients with rheumatic diseases, a quantitative synthesis of the existing evidence is lacking. This study aims to systematically evaluate the overall effects of IF on disease activity and inflammatory markers in patients with rheumatic diseases.

**Methods:**

A systematic search was conducted in PubMed, Web of Science, Embase, and The Cochrane Library for clinical studies examining intermittent fasting interventions in rheumatic diseases, with a search deadline of March 15, 2026. RevMan 5.4 software was utilized to perform a meta-analysis on the studies that met the inclusion criteria.

**Results:**

A total of seven independent studies (yielding nine datasets) were included in the analysis, comprising 471 patients with rheumatoid arthritis (RA) and 85 patients with spondyloarthritis (SpA). The results indicated that observational studies, which provide a lower level of evidence, demonstrated significant improvements in disease activity scores associated with intermittent fasting (*e.g*., Visual Analog Scale (VAS), *P* < 0.05; Disease Activity Score in 28 joints (DAS28), *P* < 0.05). Additionally, an analysis of three randomized controlled trials (RCTs) revealed that (IF) significantly reduced DAS28 scores (MD = −0.55, 95% CI [−1.09 to −0.02], *P* = 0.04).

**Conclusion:**

These findings suggest a positive effect of intermittent fasting on disease activity, as indicated by both observational studies and RCTs. However, the reliability of the conclusions drawn from RCTs is limited due to the small number of studies and considerable heterogeneity in the interventions. There is an urgent need for more rigorously designed RCTs to further validate these results.

## Introduction

Rheumatic diseases are systemic disorders characterized by immune dysregulation and inflammatory responses. These conditions involve multiple systems and are marked by a chronic course with alternating periods of disease activity and remission. They can significantly affect various tissues, including bursae, tendons, fascia, blood vessels, and nerves. Intermittent fasting (IF) is an umbrella term for various dietary patterns designed to improve health by limiting the timing of food intake within specific cycles (Varady, 2021). IF can be divided into various methods, including time-restricted eating, alternate day fasting, and periodic fasting. Existing research indicates that IF can have varying degrees of beneficial effects on multiple diseases (Reddy, Reddy & Saier, 2024).

One of the most common clinical symptoms of rheumatic diseases is joint pain caused by inflammation. Several studies indicate that IF can improve inflammation in various parts of the body, though the extent of improvement may vary (Paoli et al., 2024). Its potential regulatory mechanisms on inflammatory pathways have also garnered significant attention (Han et al., 2021). However, there is a lack of systematic quantitative evidence regarding the effectiveness of IF in the field of rheumatic diseases. Many individual studies have small sample sizes and varied intervention protocols, resulting in inconsistent conclusions. To address this gap, this study aims to comprehensively and quantitatively synthesize the existing clinical research evidence through a systematic review and meta-analysis. This aimed to elucidate the overall impact of IF on disease activity and inflammatory markers in patients with rheumatic diseases, thereby providing higher-level evidence-based medical support for this non-pharmacological therapy.

Given the limited number of available studies, we included three different fasting methods: ① Ramadan Diurnal Intermittent Fasting: Characterized by religious customs, this involves complete abstention from food and water from sunrise to sunset for about a month. ② 16:8 Intermittent Fasting: Defined as a form of Time-Restricted Feeding, where the daily eating window is limited to 8 h, with the remaining 16 h spent fasting. ③ Low-calorie fasting & Fasting after bowel cleansing: This involves a 7–10 day fast initiated after bowel cleansing, with a daily caloric intake of less than 250 kcal.

## Methods

### Review registration

This systematic review and meta-analysis were conducted and reported in accordance with the 2021 updated Preferred Reporting Items for Systematic Reviews and Meta-Analyses (PRISMA) statement (Page et al., 2021). This review was registered in the International Prospective Register of Systematic Reviews (PROSPERO), registration number: CRD420251089968.

### Data sources and search strategy

**Figure 1:**
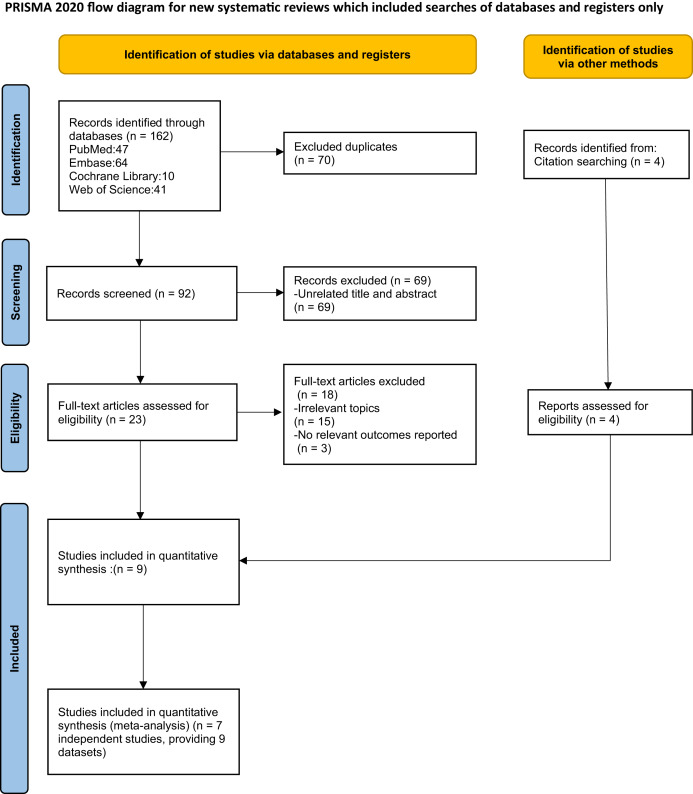
Studies’ screening process.

A systematic search was conducted across PubMed, Web of Science, Embase, and The Cochrane Library to identify studies examining the effects of IF on rheumatic diseases, with a deadline set for March 15, 2026. The primary search terms included “intermittent fasting” and various rheumatic conditions such as “rheumatism, rheumatoid arthritis, osteoarthritis, ankylosing spondylitis, and systemic lupus erythematosus.” A combination of subject headings and keywords was used. The detailed electronic search strategies for all databases are provided in Material S1. The PRISMA flow diagram of the search results is shown in Fig. 1.

### Study selection and inclusion criteria

Search records were systematically merged and duplicates eliminated utilizing EndNote 21. The screening process was performed by two independent authors (Liu and Zhang): the initial screening focused on titles and abstracts, followed by a comprehensive evaluation of full texts to exclude studies that did not conform to the research scope or failed to report the requisite outcome measures. Any discrepancies between the reviewers were adjudicated by a third author (Li). All studies included in our analysis adhered to the PICOS criteria, which are delineated as follows: (1) Population: Patients diagnosed with at least one rheumatic disease, specifically rheumatoid arthritis, ankylosing spondylitis, or psoriatic arthritis; (2) Intervention: A minimum of 7 days of IF; (3) Comparison: The study involved an IF intervention and included comparisons of pre- and post-fasting data; (4) Outcomes: The primary outcome measures selected were: ① composite scores for overall disease activity, encompassing the 28-joint Disease Activity Score (DAS28), and the Clinical Disease Activity Index (CDAI) for RA, as well as the Bath Ankylosing Spondylitis Disease Activity Index (BASDAI) for spondyloarthritis; ② the Visual Analogue Scale (VAS) for patient-reported symptoms. Secondary outcome measures included: ① morning stiffness (MS) as a specific clinical symptom; ② inflammatory biomarkers, including Erythrocyte Sedimentation Rate (ESR) and high-sensitivity C-reactive protein (hs-CRP). (5) Study Design: Included were observational studies, randomized controlled trials, and open-label studies. Excluded from the analysis were case reports, editorials, letters, expert opinions, reviews, animal studies, and articles for which the full text or original data were unavailable.

### Data extraction

Data were extracted from the included studies using a pre-specified Excel form. Two independent reviewers (Liu and Zhang) extracted the relevant information from eligible articles including the following aspects: first author, publication year, country of origin, study design, inclusion and exclusion criteria, patient age, disease type, follow-up or intervention duration, treatment protocols, sample size, outcome measures, and the mean change and standard deviations (SDs) for all aforementioned outcomes. Any disagreements that arose during this process were resolved by a third party (Li).

### Risk of bias assessment

Two researchers (Liu and Zhang) used the Newcastle-Ottawa Scale (NOS) to assess the risk of bias in cohort and case-control studies. Each NOS scale consists of a maximum total of nine points, which are distributed across three domains: four points are allocated for the selection of study groups, two points for the comparability of groups, and three points for the ascertainment of exposure or outcome. Studies achieving a total score of seven or higher were considered as high quality, scores between four and six were categorized as moderate quality, and scores of three or lower were deemed low quality. Any discrepancies among reviewers were resolved by a third party (Li). Studies were subsequently rated as good, fair, or poor quality based on their respective total NOS scores.

### Data synthesis and statistical analysis

This study employed RevMan 5.4 software for conducting the meta-analysis and generating funnel plots. The mean and SD of the changes were utilized to assess the effect. In cases where studies did not provide the SD of the change, it was calculated using the changes in the concentration of each variable throughout the study (Hozo, Djulbegovic & Hozo, 2005). Considering that the original studies did not report the correlation coefficients between pre- and post-intervention measurements, a conservative moderate correlation value (Corr = 0.5) was assumed for the primary analysis. In the sensitivity analyses, lower (Corr = 0.3) and higher (Corr = 0.8) correlation coefficients were applied to recalculate and pool the effect estimates, in order to assess the robustness of the results. For all analyses, we calculated the mean difference (MD) with a 95% confidence interval (CI) to facilitate comparison across studies that employed different measurement scales. We utilized a random-effects model to account for variations between studies and to determine the overall effect size. The I^2^ statistic was employed to determine heterogeneity, with an I^2^ value > 50% indicating significant heterogeneity among the studies. An influence analysis was conducted to ensure that the overall effect size was not unduly influenced by any single study. We statistically assessed publication bias using the PET-PEESE test and funnel plots, with a *P*-value < 0.05 was considered statistically significant. To address the risk of bias introduced by different study designs, we plan to conduct a subgroup analysis to separately combine the results of RCT and observational studies. Additionally, we will conduct a subgroup analysis based on study design to further clarify the impact of the intervention, distinguishing between observational studies and RCTs (observational studies *vs*. RCTs).

## Results

### Characteristics of included studies

**Figure 2:**
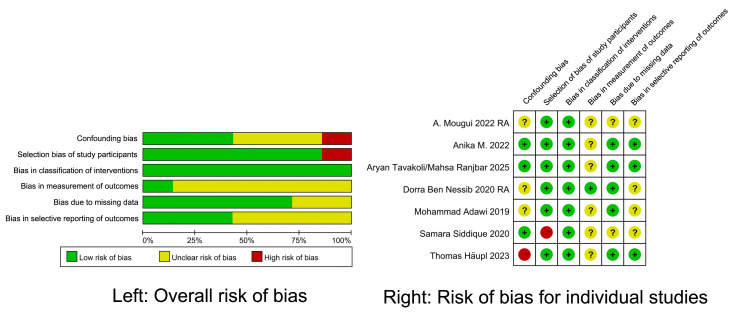
Results of risk of bias assessment for the included studies.

A total of 162 records were identified through database searches. After duplicates were removed and titles and abstracts were screened, 23 reports from the databases and four additional records from manual citation searches were evaluated for full-text eligibility. Ultimately, nine articles met the inclusion criteria, with publication years ranging from 2019 to 2025. The basic characteristics of these included studies are presented in Table 1, while the results of the risk of bias assessment are displayed in Fig. 2. Among the included studies, five were observational studies (Adawi et al., 2019; Ben Nessib et al., 2020, 2022; Mougui, Tebaa & El Bouchti, 2023; Siddique et al., 2020), one was an open-label study (Haupl et al., 2023), and three were RCTs (Hartmann et al., 2022; Ranjbar et al., 2025; Tavakoli et al., 2025). These studies originated from seven countries, with the following distribution: Italy (*n* = 1) (Adawi et al., 2019), Iran (*n* = 2) (Ranjbar et al., 2025; Tavakoli et al., 2025), Tunisia (*n* = 2) (Ben Nessib et al., 2020, 2022), Germany (*n* = 1) (Haupl et al., 2023), Morocco (*n* = 1) (Mougui, Tebaa & El Bouchti, 2023), Pakistan (*n* = 1) (Siddique et al., 2020), and France (*n* = 1) (Hartmann et al., 2022). The nine included articles corresponded to seven independent clinical studies, as some studies led to multiple publications while others reported outcomes for different disease populations. After consolidation, six studies focused on IF in RA, with a total sample size of 471 participants, while four studies examined IF in SpA, comprising 85 participants. The duration of fasting varied between 7 and 30 days, and the intervention modalities included intermittent diurnal fasting, 16:8 time-restricted eating, and Ramadan diurnal IF.

**Table 1:** Basic characteristics of the included studies.

Study	Country	Disease type	Study design	N	Age (years)	Intervention model	Fasting mode	Duration	Outcome measures
Adawi et al. (2019), Damiani et al. (2019)	Italy	PsA	Observational study	37	43.32 ± 7.81	Intermittent diurnal fasting	Fasting from sunrise to sunset	30 days	⑥⑧
Ranjbar et al. (2025), Tavakoli et al. (2025)	Iran	RA	RCT	44	56.41 ± 5.44	16:8 intermittent fasting	16-h fasting period alternating with an 8-h eating window	56 days	②③④⑦⑧
Ben Nessib et al. (2020), (2022)	Tunisia	RA	Observational study	30	57.5 ± 10.9	Ramadan diurnal intermittent fasting	Fasting from sunrise to sunset, with refeeding at night	30 days	①③④⑦⑧
	Tunisia	SpA	Observational study	26	47 ± 12.6			30 days	①④⑥⑦⑧
Haupl et al. (2023)	Germany	RA	Open-label study	20	51.05 ± 16.96	Fasting after bowel cleansing	Fasting for 7–10 days after bowel cleansing	7–10 days	③⑤
Mougui, Tebaa & El Bouchti (2023)	Morocco	RA	Observational study	49	NR	Ramadan diurnal intermittent fasting	Fasting from sunrise to sunset, with refeeding at night	30 days	①②③④⑤
	Morocco	SpA	Observational study	22	NR			30 days	①⑥
Siddique et al. (2020)	Pakistan	RA	Observational study	120	37 (NR)	Ramadan diurnal intermittent fasting	Fasting from sunrise to sunset, with refeeding at night	30 days	③
Hartmann et al. (2022)	France	RA	RCT	27	52.8 ± 6.8	Low-calorie fasting	7-day fast followed by an 11-week plant-based diet	84 days	②③⑤⑦⑧

**Note:**

RCT, Randomized controlled trial; 16:8 intermittent fasting: Alternating between a 16-h fasting period and an 8-h eating window; Fasting after bowel cleansing: A 7–10 day fast following bowel cleansing, during which daily intake is less than 250 kcal from vegetable juice, sugar-free tea, or water (≥2 L/day); Low-calorie fasting: Daily energy intake of 300–350 kcal from vegetable juices and vegetable soup. ① VAS: Visual Analogue Scale; ②CDAI: Clinical Disease Activity Index; ③ DAS28: 28-joint Disease Activity Score; ④ MS: Morning Stiffness; ⑤ SDAI: Simplified Disease Activity Index; ⑥BASDAI: Bath Ankylosing Spondylitis Disease Activity Index; ⑦ ESR: Erythrocyte Sedimentation Rate; ⑧ hs-CRP: high-sensitivity C-reactive Protein.

### Meta-analysis results

A total of seven independent clinical studies were included in the meta-analysis, providing nine distinct datasets for quantitative synthesis. Of these, two studies assessed both RA and SpA (Ben Nessib et al., 2020, 2022; Mougui, Tebaa & El Bouchti, 2023), providing pre- and post-fasting data for each, which were treated as independent studies in the analysis.

#### Subgroup analysis of observational studies

In the subgroup analysis of observational studies, a significant improvement was observed for both VAS (MD = −1.71 [−2.6, −0.82], *P* < 0.05) and DAS28 (MD = −1.05 [−1.36, −0.73], *P* < 0.05). The remaining outcomes did not achieve statistical significance, and high heterogeneity was observed, as detailed in Table 2. Notably, while the point estimates for BASDAI, MS, and ESR indicated a clear trend, their 95% confidence intervals were close to the null value, precluding definitive conclusions.

**Table 2:** Subgroup analysis of observational studies.

Outcome measure	No. of studies	Participants (n)	Heterogeneity (I^2^, %)	MD (95% CI)	*P*-value
VAS	4	127	78%	−1.71 [−2.60 to −0.82]	<0.05
DAS28	4	225	67%	−1.05 [−1.36 to −0.73]	<0.05
BASDAI	3	79	97%	−1.19 [−2.66 to 0.27]	0.11
MS	3	105	90%	−16.76 [−37.14 to 3.62]	0.11
ESR	2	56	73%	−9.51 [−19.51 to 0.48]	0.06
hs-CRP	3	93	39%	−1.61 [−4.26 to 1.03]	0.23

**Note:**

Results were pooled using a random-effects model.

#### Subgroup analysis of RCTs

In the subgroup analysis restricted to RCTs (two independent studies involving three publications, totaling 94 participants), IF was associated with a significant reduction in DAS28 (MD = −0.55, 95% CI [−1.09 to −0.02], *P* = 0.04; I^2^ = 0%). However, although CDAI also showed a decreasing trend (MD = −3.71, 95% CI [−11.35 to 3.93], *P* = 0.34; I^2^ = 66%), this change did not reach statistical significance (Table 3). ESR showed a clear trend toward improvement, exhibiting good homogeneity (MD = −5.94 [−12.52, 0.64], P = 0.08; I^2^ = 0%) However, the 95% confidence interval included the null value, indicating that it did not achieve statistical significance, likely due to the small sample size. In contrast, no significant effect was found for hs-CRP (MD = −2.71 [−8.76, 3.33], *P* = 0.38), and substantial heterogeneity was detected (I^2^ = 92%).

**Table 3:** Subgroup analysis of RCTs.

Outcome measure	No. of RCTs	Participants (*n*)	Heterogeneity (I^2^, %)	MD (95% CI)	*P*-value
DAS28	2	94	0%	−0.55 [−1.09 to −0.02]	0.04
CDAI	2	94	66%	−3.71 [−11.35 to 3.93]	0.34
ESR	2	94	0%	−5.94 [−12.52 to 0.64]	0.08
hs-CRP	2	94	92%	−2.71 [−8.76 to 3.33]	0.38

**Note:**

Results were pooled using a random-effects model.

### Sensitivity analysis

Sensitivity analyses were conducted on the outcome measures in the observational studies that required estimating standard deviations. When the assumed correlation coefficient for these calculations was varied from 0.5 to 0.3 and 0.8, the re-pooled meta-analysis results revealed no significant changes in either the magnitude or the statistical significance of the combined effect sizes. These findings suggest that the conclusions drawn from the observational studies are robust and not influenced by the choice of correlation coefficient used for estimating standard deviations.

## Discussion

Current treatment strategies for rheumatic diseases have evolved over many years and can be broadly categorized into three types: pharmacological therapy, surgical treatment, and basic therapy. Pharmacological treatment typically involves the use of anti-rheumatic drugs, analgesics, and non-steroidal anti-inflammatory drugs (NSAIDs), but these options often face challenges related to limited efficacy and potential side effects. When conservative treatments prove ineffective, surgical intervention may be considered to alleviate patients’ symptoms; however, the applicability and long-term prognosis of invasive methods are often limited. Basic therapy, on the other hand, emphasizes a patient-centered approach, encouraging individuals to improve their lifestyle to alleviate disease symptoms (Burmester et al., 2017). As a novel fasting protocol, IF is gaining increasing recognition and has been confirmed as a safe and effective way to manage weight and improve metabolic health (Varady et al., 2024); its potential in controlling inflammation and pain is still under exploration.

Despite rapid advancements in rheumatology research in recent decades, the etiology of rheumatic diseases is still not fully understood. Currently, it is recognized that factors such as diet, gut microbiota, metabolism, and BMI influence rheumatic diseases to varying degrees (Diaz-Gonzalez & Hernandez-Hernandez, 2023). Fasting has a significant impact on diet, gut microbiota (Secor & Carey, 2016; Thaiss et al., 2014), metabolism (Lessan & Ali, 2019), BMI, as well as levels of inflammation and pain (Michalsen, 2024). Animal studies have demonstrated specific effects of fasting therapy. In mouse models, fasting suppressed T-cell activation, proliferation, differentiation, and cytokine production in autoimmune conditions, leading to a significant alleviation of symptoms (Wang et al., 2024). Another study demonstrated that fasting had no adverse effects on mice with experimental autoimmune encephalomyelitis (EAE). In fact, it not only reduced disease activity but also suppressed the production of IFN-γ, TNF-α, and IL-10 (Razeghi Jahromi et al., 2016).

To enhance the specificity of this study, subgroup analyses were conducted separately for observational studies and RCTs. The results indicated that IF was associated with improvements in disease activity across studies of varying levels of evidence. However, it is important to acknowledge that the significant effects observed in this meta-analysis may, in part, be attributable to the inherent confounding factors present in observational studies, while the number of RCTs included was limited (*n* = 3). Therefore, these findings should be interpreted and generalized with caution. Given the relatively small number of studies included for each outcome (*e.g*., *n* ≤ 4), a meaningful assessment of publication bias could not be performed. Similarly, an analysis of the underlying reasons for heterogeneity was not performed due to the limited number of included studies (<10). This review did not undertake a formal certainty assessment of the GRADE evidence, owing to the limited number of studies and the heterogeneity of the results. The insufficient number of studies rendered both visual inspection of funnel plots and related statistical tests unreliable; therefore, these results were not reported in this study.

The subgroup analysis of RCTs also presented several notable limitations. First, the two independent RCT studies (represented by three publications) employed differing intervention methods—one utilized a 16:8 IF regimen, while the other implemented a low-calorie diet following bowel cleansing—which may have contributed to distorted results and increased heterogeneity. Second, the total sample size was limited, thereby reducing the statistical power of the analysis. Furthermore, although both RCTs independently demonstrated a discernible trend toward improvement in disease activity with IF, the reductions in inflammatory biomarkers were not statistically significant.

This finding is inconsistent with existing meta-analyses on other diseases, one of which reported that IF significantly improved inflammatory profiles (Lu et al., 2025), which should theoretically yield even more pronounced improvements in rheumatic diseases driven by immune-mediated inflammation and pain. This suggests that IF is a promising area for future exploration and highlights the need for more, larger-scale, high-quality RCTs. Notably, the current consensus suggests a correlation between MS and scores like DAS28 (Fanta et al., 2014; Krijbolder et al., 2022). Our analysis revealed that fasting significantly affected VAS and DAS28, but its impact on MS was not significant. This suggests that fasting may have a greater influence on objective indicators such as joint swelling and pain than on the subjective symptom of MS. The assessment of MS is highly subjective, which could also contribute to the variability of the results. Consequently, the degree of improvement in inflammation was less clear.

Furthermore, we observed high statistical heterogeneity (high I^2^ values) for several outcome measures, primarily due to differences among the included studies on multiple levels. These differences include: ① diversity of disease types (including both RA and SpA); ② various intervention protocols (covering Ramadan fasting, 16:8 fasting, and low-calorie fasting); and ③ variations in patient baseline characteristics (including disease duration and baseline medications). All these factors could influence the overall effects of the interventions. Furthermore, improvements in clinical symptoms of other rheumatic diseases due to IF have been noted in literature beyond this analysis. A study in Kuwait showed that IF significantly improved the PASI score in patients with stable chronic plaque psoriasis, with the difference being statistically significant (*P* = 0.001) (Almutairi & Shaaban, 2022), which indirectly corroborates the results of this study.

Despite the need for more homogeneous studies to accurately estimate the effect size due to significant heterogeneity, our findings provide strong evidence-based support for IF as a potential non-pharmacological adjunctive therapy. Based on the results of this study, we believe that more well-designed, larger-sample, high-quality RCTs on IF are needed to strengthen the evidence base. Future research should focus on specific fasting protocols, such as 16:8 fasting or Ramadan IF, targeting particular rheumatic diseases to minimize clinical heterogeneity. Additionally, long-term follow-up studies are essential for assessing the long-term effects, safety, and patient adherence to IF.

The included studies indicate that IF may positively affect rheumatic diseases through multiple pathways. Furthermore, the incidence of adverse effects from fasting therapy is low, with the consequences of these adverse events generally being mild. A retrospective study on water-only fasting revealed that most adverse events were mild, with serious adverse events occurring in only 0.002% of patients (Finnell et al., 2018). In clinical practice, patients often face mandatory drug discontinuation due to liver or kidney damage (Aletaha & Kerschbaumer, 2017), which remains a significant challenge for physicians within the current treatment paradigms. In such necessary situations, improving symptoms through lifestyle changes is a viable option. It is important to note that current EULAR and French Society of Rheumatology guidelines do not recommend fasting as a routine treatment (Fautrel et al., 2024; Smolen et al., 2023). This is primarily attributable to the scarcity of high-quality evidence. The findings of our study contribute new evidence pertinent to future guideline updates; however, they necessitate validation through larger-scale RCTs. Current EULAR guidelines predominantly advocate for the Mediterranean diet for patients, as research has demonstrated its advantages concerning disease activity and cardiovascular comorbidities in RA patients (Smolen et al., 2023). IF is a dietary pattern that merits consideration, particularly for patients who have developed drug resistance or have contraindications to medications. This makes it a viable non-pharmacological treatment option.

## Conclusion

This systematic review and meta-analysis, encompassing nine publications representing seven independent clinical studies (yielding nine datasets), systematically evaluated the impact of IF on disease activity and inflammation levels in patients with rheumatic diseases. The meta-analysis of observational studies demonstrated that IF significantly reduced VAS and DAS28 scores, indicating a beneficial effect in alleviating pain, joint swelling, and overall disease status. Although a trend towards improvement was observed for inflammatory markers, the pooled analysis did not achieve statistical significance. Based on the currently limited high-quality evidence from randomized controlled trials (RCTs), IF may also contribute to the enhancement of disease activity in RA. However, in light of the substantial limitations identified, the robustness of this conclusion remains questionable, underscoring the urgent need for rigorously designed and well-standardized RCTs to further validate these findings.

## Supplemental Information

10.7717/peerj.21185/supp-1 Supplemental Information 1 PRISMA checklist.

10.7717/peerj.21185/supp-2 Supplemental Information 2 Observational study.

10.7717/peerj.21185/supp-3 Supplemental Information 3 RCT data.

10.7717/peerj.21185/supp-4 Supplemental Information 4 Sensitivity analysis.

10.7717/peerj.21185/supp-5 Supplemental Information 5 Detailed Search Strategies.

## Additional Information and Declarations

### Data Availability

The following information was supplied regarding data availability:

This is a Systematic Review/Meta-analysis.
