# Supplementary material for: Intermittent fasting for rheumatic diseases: a systematic review and meta-analysis of conflicting evidence from observational studies and randomized controlled trials
Source: PeerJ. 2026 Apr 27;14:e21185. doi: 10.7717/peerj.21185 (PMC13131347; doi:10.7717/peerj.21185)
Supplement: Supplemental Information 5 [file peerj-14-21185-s005.docx]

- **Pubmed: ("Intermittent Fasting"[Mesh] OR "intermittent fasting"[tw] OR "Time Restricted Feeding"[tw] OR "Time Restricted Fasting"[tw] OR "Time Restricted Eating"[tw] OR "Meal Skipping"[tw] OR "intermittent fasting"[tiab:~3] OR "Time Restricted Feeding"[tiab:~3] OR "Time Restricted Fasting"[tiab:~3] OR "Time Restricted Eating"[tiab:~3] OR "Meal Skipping"[tiab:~3]) AND ("Rheumatic Diseases"[Mesh] OR "Rheumatic Diseases"[tw] OR "Rheumatic Diseases"[tw] OR "rheumatism" OR "rheumatoid arthritis" OR "rheumatoid" OR "arthritis" OR "osteoarthritis" OR "ankylosing spondylitis" OR "Lupus Erythematosus, Systemic"[Mesh] OR "SLE"[tw] OR "systemic lupus erythematosus")**
- **Embase Strategy: ( 'intermittent fasting'/exp OR 'intermittent fasting':ti,ab OR 'time restricted feeding':ti,ab OR 'time restricted fasting':ti,ab OR 'time restricted eating':ti,ab OR 'meal skipping':ti,ab OR (intermittent NEAR/3 fasting):ti,ab OR ('time restricted' NEAR/3 feeding):ti,ab OR ('time restricted' NEAR/3 fasting):ti,ab OR ('time restricted' NEAR/3 eating):ti,ab OR (meal NEAR/3 skipping):ti,ab ) AND ( 'rheumatic disease'/exp OR 'rheumatism':ti,ab OR 'rheumatoid arthritis':ti,ab OR 'rheumatoid':ti,ab OR 'arthritis':ti,ab OR 'osteoarthritis':ti,ab OR 'ankylosing spondylitis':ti,ab OR 'systemic lupus erythematosus'/exp OR 'sle':ti,ab OR 'systemic lupus erythematosus':ti,ab )**
- **Web of Science Strategy: TS=( ( "intermittent fasting" OR "time restricted feeding" OR "time restricted fasting" OR "time restricted eating" OR "meal skipping" OR (intermittent NEAR/3 fasting) OR ("time restricted" NEAR/3 feeding) OR ("time restricted" NEAR/3 fasting) OR ("time restricted" NEAR/3 eating) OR (meal NEAR/3 skipping) ) AND ( "rheumatism" OR "rheumatoid arthritis" OR "rheumatoid" OR "arthritis" OR "osteoarthritis" OR "ankylosing spondylitis" OR "systemic lupus erythematosus" OR "SLE" OR "rheumatic diseases" ) )**
- **The Cochrane Library Strategy: ( [mh "Intermittent Fasting"] OR "intermittent fasting":ti,ab,kw OR "time restricted feeding":ti,ab,kw OR "time restricted fasting":ti,ab,kw OR "time restricted eating":ti,ab,kw OR "meal skipping":ti,ab,kw OR (intermittent NEAR/3 fasting):ti,ab,kw OR ("time restricted" NEAR/3 feeding):ti,ab,kw OR ("time restricted" NEAR/3 fasting):ti,ab,kw OR ("time restricted" NEAR/3 eating):ti,ab,kw OR (meal NEAR/3 skipping):ti,ab,kw ) AND ( [mh "Rheumatic Diseases"] OR "rheumatism":ti,ab,kw OR "rheumatoid arthritis":ti,ab,kw OR "rheumatoid":ti,ab,kw OR "arthritis":ti,ab,kw OR "osteoarthritis":ti,ab,kw OR "ankylosing spondylitis":ti,ab,kw OR [mh "Lupus Erythematosus, Systemic"] OR "sle":ti,ab,kw OR "systemic lupus erythematosus":ti,ab,kw )**
